# Clinical characterization of "visual snow" (Positive Persistent Visual Disturbance)

**DOI:** 10.1186/1129-2377-14-S1-P132

**Published:** 2013-02-21

**Authors:** CJ Schankin, F Maniyar, J Hoffmann, D Chou, PJ Goadsby

**Affiliations:** 1 UCSF Headache Center, USA

## Background

Visual snow has a major impact on patients' quality of life. They describe persistent, dynamic, black & white tiny dots in the entire visual field (main criterion). Additional symptoms might be present. The literature on this condition is scarce [1-3] and there is some confusion with persistent migraine aura or LSD-flashback.

## Objective

To describe the clinical phenotype of patients with visual snow.

## Methods

Preliminary additional criteria for visual snow were generated retrospectively based on an internet-survey. By using telephone interviews, we re-tested these criteria prospectively and collected further information.

## Results

The survey-data of 120 patients with self-reported visual snow was reviewed to generate preliminary criteria. These were re-tested by interviewing 115 patients. The main criterion (black and white visual snow) was met by 57 patients. Additional visual symptoms were excessive floaters (84%), persistent after-images (83%), "hard time seeing at night" (65%), "little cells that travel on a wiggly path" (77%), photophobia (70%), "moving objects leave trails" (56%), "swirls, clouds or waves with eyes closed" (51%), and bright flashes (51%). Requiring at least one or three of these additional criteria reduced the sensitivity by 2% and 7%. No patient described the visual symptoms consistent with persistent visual aura in migraine. A history of migraine was seen in 54%, with 35% having typical migraine with aura. None of the patients noted intake of illicit drugs prior the onset of visual snow. All ophthalmology tests were non-contributory.

## Conclusions

(i) Visual snow is a unique clinical syndrome. The main criterion (visual snow) is almost always associated with at least three additional criteria. (ii) The visual symptoms are distinct from migraine with aura. (iii) The high prevalence of history of migraine (with aura) points to a susceptibility for visual snow in patients with migraine. (iv) Intake of illicit drugs and ophthalmological diseases may not be of pathophysiological relevance.
